# Perioperative analgesia after intrathecal fentanyl and morphine or morphine alone for cesarean section: A randomized controlled study

**DOI:** 10.1097/MD.0000000000008892

**Published:** 2017-12-01

**Authors:** Wojciech Weigl, Andrzej Bieryło, Monika Wielgus, Świetlana Krzemień-Wiczyńska, Marcin Kołacz, Michał J. Dąbrowski

**Affiliations:** a First Department of Anesthesiology and Intensive Care, Medical University of Warsaw, Warsaw, Poland; b Anesthesiology and Intensive Care, Department of Surgical Sciences, Uppsala University, Akademiska Hospital, Uppsala, Sweden; c Department of Anesthesiology and Intensive Care, Centre of Postgraduate Medical Education, Gruca Orthopedic and Trauma Teaching Hospital, Otwock; d Institute of Computer Science, Polish Academy of Sciences, Warsaw, Poland.

**Keywords:** acute opioid tolerance, hydrophilic and lipophilic opioids, patient-controlled analgesia (PCA), post-caesarean pain management, spinal anesthesia, spinal opioids, superiority and noninferiority trial

## Abstract

**Objectives:**

Intrathecal morphine is used in the postoperative management of pain after caesarean section (CS), but might not be optimal for intraoperative analgesia. We hypothesized that intrathecal fentanyl could supplement intraoperative analgesia when added to a local anesthetic and morphine without affecting management of postoperative pain.

**Methods:**

This prospective, randomized, double-blind, parallel-group study included 60 parturients scheduled for elective CS. Spinal anesthesia consisted of bupivacaine with either morphine 100 μg (M group), or fentanyl 25 μg and morphine 100 μg (FM group). The frequency of intraoperative pain and pethidine consumption in the 24 hours postoperatively was recorded.

**Results:**

Fewer patients in the FM group required additional intraoperative analgesia (*P* < .01, relative risk 0.06, 95% confidence interval [CI] 0.004–1.04). The FM group was noninferior to the M group for 24-hour opioid consumption (95% CI −10.0 mg to 45.7 mg, which was below the prespecified boundary of 50 mg). Pethidine consumption in postoperative hours 1 to 12 was significantly higher in the FM group (*P* = .02). Postoperative nausea and vomiting (PONV) were more common in the FM group (*P* = .01). Visual analog scale scores, effective analgesia, Apgar scores, and rates of pruritus and respiratory depression were similar between the groups.

**Conclusions:**

Intrathecal combination of fentanyl and morphine may provide better perioperative analgesia than morphine alone in CS and could be useful when the time from anesthesia to skin incision is short. However, an increase in PONV and possible acute spinal opioid tolerance after addition of intrathecal fentanyl warrants further investigation using lower doses of fentanyl.

## Abstract 2

Supplemental Digital Content is available in the text

## 1 Introduction

Multimodal analgesia is established in the management of pain after cesarean section (CS), and different regimens are being tested.^[1]^ However, the most popular strategies include intrathecal opioids, among which morphine, a hydrophilic opioid, is recognized as a gold standard.^[2]^ It has been shown that morphine can provide postoperative analgesia for up to 24 hours,^[3]^ and a dose of up to 100 μg is relatively safe in patients undergoing CS. However, intrathecal morphine has a slow onset of action of, approximately 30 minutes.^[4]^ In many centers, CS is started before the full onset of action of morphine. In this situation, spinal anesthesia is based solely on administration of local anesthetics. This could be the reason why many studies have reported that intraoperative pain occurs in a considerable number of patients during CS, even in those in whom spinal morphine was added to a local anesthetic agent. ^[5,6]^ This comparatively high rate of intraoperative pain is in fact similar to that observed when local anesthetics are used in spinal anesthesia without any opioid added.^[7–12]^ One solution to the slow onset of action of morphine might be addition of lipophilic opioids that act more rapidly. Intrathecal fentanyl may improve intraoperative analgesia and provide effective postoperative analgesia when it is most needed after CS.^[13]^ However, although intrathecal fentanyl is beneficial in the early postoperative period, it seems not to be optimal for long-lasting analgesia.

In the search for the best components of analgesia, which would include both a rapid onset and a long-lasting duration of action, a combination of intrathecal adjuvants has been proposed.^[14]^ The pharmacological properties of lipophilic and hydrophilic intrathecal opioids suggest that they could act in a complementary manner and increase the quality of intraoperative analgesia and the duration of postoperative analgesia. However, despite these favorable pharmacodynamic properties, there is limited published research on intrathecal use of a combination of hydrophilic and lipophilic opioids for women undergoing CS, even though this is common practice in some countries.^[15]^ Further, the conclusions of previous studies have been inconsistent,^[16]^ and some have reported acute opioid tolerance after mixing intrathecal opioids.^[6]^ Given the lack of agreement regarding optimal perioperative analgesia in CS, reports showing that existing analgesia regimens are inadequate,^[17]^ and the high frequency with which CS is performed worldwide,^[18]^ we undertook this study to determine if a combination of fentanyl and morphine would provide better perioperative analgesia than morphine alone. A secondary aim was to investigate the safety of intrathecal opioids, including the potential for acute spinal opioid tolerance.

## 2 Methods

### 2.1 Study design and population

The study was conducted in the Department of Obstetrics and Gynecology at the Medical University of Warsaw in Poland. Sixty parturients aged 18 to 45 years (American Society of Anesthesiologists physical status I or II, >36 weeks’ gestation) and scheduled for elective CS under spinal anesthesia were randomized in an allocation ratio of 1:1 to participate in this prospective, double-blind, parallel-group study. The study received ethical approval from the Ethics Committee of the Medical University of Warsaw (KB/60/2009) and was conducted in accordance with the principles laid out in the Declaration of Helsinki and national regulations. All study participants gave written informed consent after receiving detailed verbal and written explanations regarding the aims and methods of the study during a preoperative visit. The patients were monitored regularly to assess whether our postoperative pain management protocol was satisfactory, and to identify any serious safety concerns. No changes to the study protocol were made after the trial commenced. The study adheres to the consolidated standards of reporting trials statement guidelines (CONSORT).

### 2.2 Anesthesia

This trial was a continuation of our previous study on intrathecal opioids in CS.^[13]^ The same study design (randomization, allocation, and blinding methods) and anesthesia procedures (including lumbar puncture, bupivacaine doses, and pain control regimen after CS) were used. Briefly, spinal anesthesia was performed as described previously^[13]^ using hyperbaric bupivacaine 0.5%. Bupivacaine was supplemented with morphine 100 μg either alone (M group) or in combination with fentanyl 25 μg (FM group). The fentanyl dose was selected on the recommendation of Hamber and Viscomi.^[19]^ The 100-μg dose of morphine was selected on the basis of a literature search, showing that a dose lower that 75 to 100 μg is not effective for management of postoperative pain,^[20,21]^ and that higher doses do not have a better analgesic effect and are associated with an increased incidence of side effects.^[21–24]^ The patients were randomized to the M group or the FM group using sealed, sequentially numbered envelopes prepared according to a computer-generated permuted-block randomization list. The participating anesthetist, patients, and postoperative staff were blinded to group allocation. Sensory block was assessed bilaterally by loss of cold sensation.

In addition to a standard pain control regimen (paracetamol and ketoprofen, intravenously [i.v.]), pethidine (meperidine) was delivered i.v. via patient-controlled analgesia (PCA; dose on demand, 10 mg; lockout interval, 10 minutes; 4-hour limit, 1.5 mg/kg; no continuous infusion, clinician bolus 10 mg).

### 2.3 Outcome measures

The anesthetist monitored the patients for intraoperative pain using a visual analog scale (VAS; 0, no pain; 10, worst imaginable pain). If the VAS exceeded 3, rescue analgesics (ketamine 10 mg i.v. before delivery and fentanyl 100 μg after delivery) were administered. Vital signs (heart rate, noninvasive arterial blood pressure, respiratory rate, oxygen saturation) were also monitored. Neonatal Apgar scores were determined at 1, 3, and 5 minutes after birth. Total hourly pethidine consumption and time to first use of PCA (effective analgesia) were recorded during the postoperative period. Vital signs and VAS scores on movement (coughing or breathing deeply) were recorded upon arrival in the postoperative unit and at 2, 4, 6, 8, 12, 16, 20, and 24 hours after induction of spinal anesthesia.

Opioid side effects, including postoperative nausea and vomiting (PONV), pruritus, oversedation, and respiratory depression, were recorded postoperatively. Oversedation was deemed to have occurred when there was no recovery of consciousness in response to a loud auditory stimulus (Ramsay Sedation Scale score 5–6). Respiratory depression was defined as a respiratory rate <8 breaths/min or arterial oxygen saturation <90.

### 2.4 Statistical analysis

The primary study endpoints were frequency of intraoperative pain (VAS score >3, resulting in administration of additional analgesia) and total pethidine consumption in the first 24 hours postoperatively. Exploratory endpoints included total pethidine consumption in the postoperative time intervals of 1 to 12 and 13 to 24 hours, effective analgesia, and VAS score. The secondary endpoints were opioid side effects, hemodynamic changes, and Apgar scores.

We based our clinical decision rule on demonstrating superiority with regard to frequency of intraoperative pain and noninferiority in total pethidine consumption in the 24 hours postoperatively for the FM group (new treatment) when compared with the M group (standard treatment).

Assuming the incidence of intraoperative pain to be 25% in the M group (see “Discussion” section) and 0% in the FM group, as documented previously,^[25–28]^ we calculated that the sample size for the superiority trial would need to be 27 in each group, with 80% power at a significance level of 5%.

To determine a prespecified noninferiority margin (*d*) for 24-hour pethidine consumption and the sample size in our noninferiority trial, we drew on the results of the study by Cohen et al^[29]^ for the M group and data from our own previous study for a placebo (no intrathecal opioids) group.^[13]^ The postoperative pain management regimens in both these studies (use of pethidine and a nonsteroidal anti-inflammatory drug) best resembled our present protocol. We assumed *d* to be 30% of the mean difference in effect between the placebo group and the M group (206 mg − 39 mg = 169 mg), which is 50 mg (by statistical reasoning). This value is also equivalent to half of 1 maximal recommended intramuscular dose of pethidine used in the management of post-CS pain, which we would consider to be a clinically meaningful difference (by clinical reasoning). We would declare noninferiority of the new treatment (FM group) with respect to the standard treatment (M group) if the upper bound of the 1-sided 95% confidence interval (CI) of the difference in means of 24-hour pethidine consumption between groups was <50 mg. Assuming the mean 24-hour pethidine consumption in the M group to be 39 mg (standard deviation [SD] 49 mg)^[29]^ and the type I and type II error to be 2.5% and 20%, respectively, we estimated that a sample size of 16 patients would be required in each group. Considering potential dropouts, we decided to enroll 30 patients per group. Correction for multiplicity in analysis of the multiple primary endpoints was not necessary because we used a gatekeeping testing approach,^[30]^ whereby we first analyzed the superiority trial. Only when the null hypotheses in the superiority trial had been rejected could we perform the noninferiority analysis, otherwise we would not be able to demonstrate any clinical benefit of combination of intrathecal fentanyl and morphine according to our clinical decision rule.

The data distribution was assessed by the Shapiro-Wilk test for normality. According to the obtained results, we used independent-samples parametric *t* test or non-parametric Wilcoxon rank test. Differences in frequency and proportions were examined using the chi-square test. The statistical analysis was performed in the R environment (R Foundation for Statistical Computing, Vienna, Austria).

## 3 Results

### 3.1 Patient characteristics

**Figure 1:**
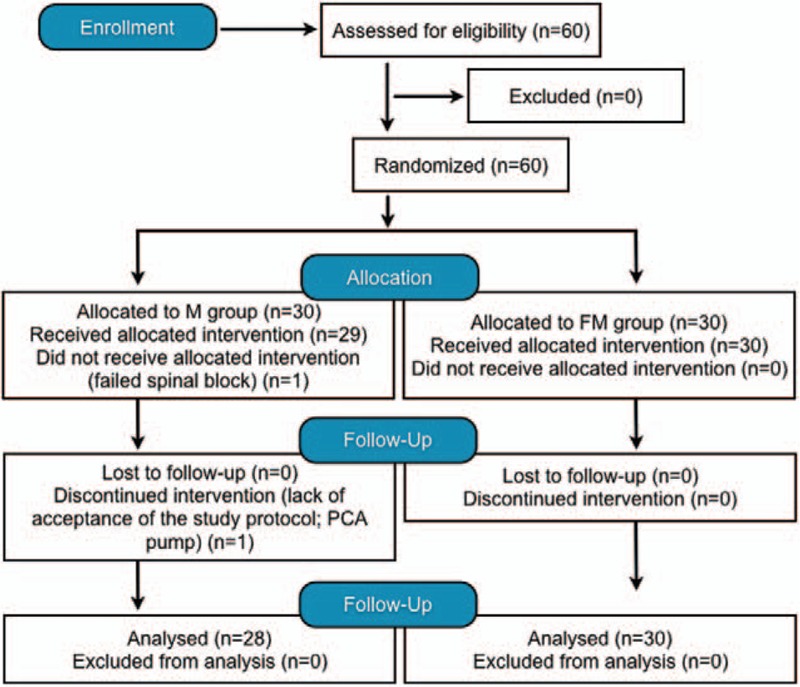
CONSORT study flow chart. CONSORT = consolidated standards of reporting trials.

Two of the 60 patients enrolled in this study between May and October 2010 (both in the M group) were excluded, one because of unsuccessful spinal blockade and the other because of lack of acceptance of the study protocol. Fifty-eight patients completed the study according to the protocol, and none was lost to follow-up. Finally, 28 parturients in the M group and 30 in the FM group were analyzed per protocol. Figure 1 shows the allocation of patients to the study groups. No differences in individual characteristics were found between the groups (Table 1). The time from induction of anesthesia to skin incision was about 10 minutes and did not differ between the groups.

**Table 1:**
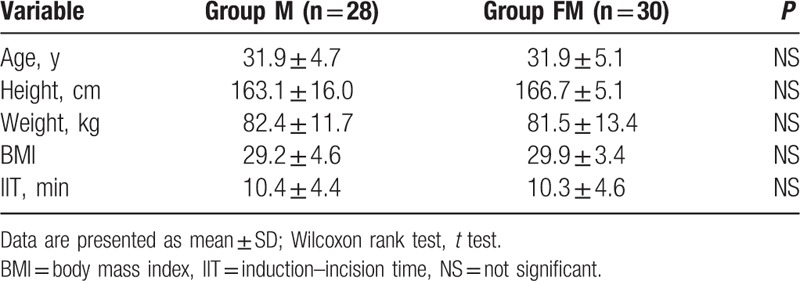
Baseline patient characteristics.

### 3.2 Primary outcome

**Figure 2:**
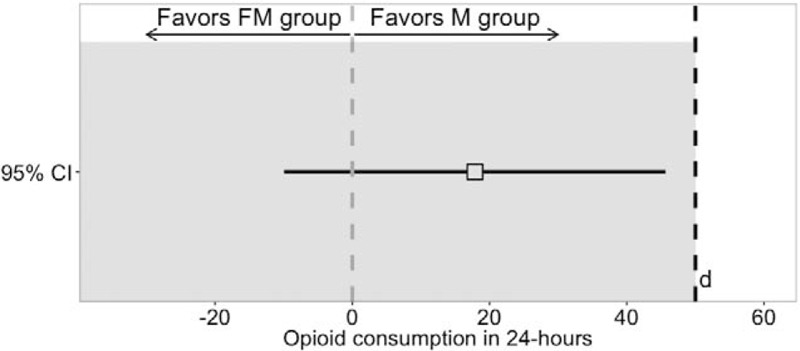
Difference in means and 95% CI of 24-hour pethidine consumption between MF and M groups in milligrams. The shaded area represents the noninferiority region; d indicates the prespecified noninferiority margin. CI = confidence interval.

**Figure 3:**
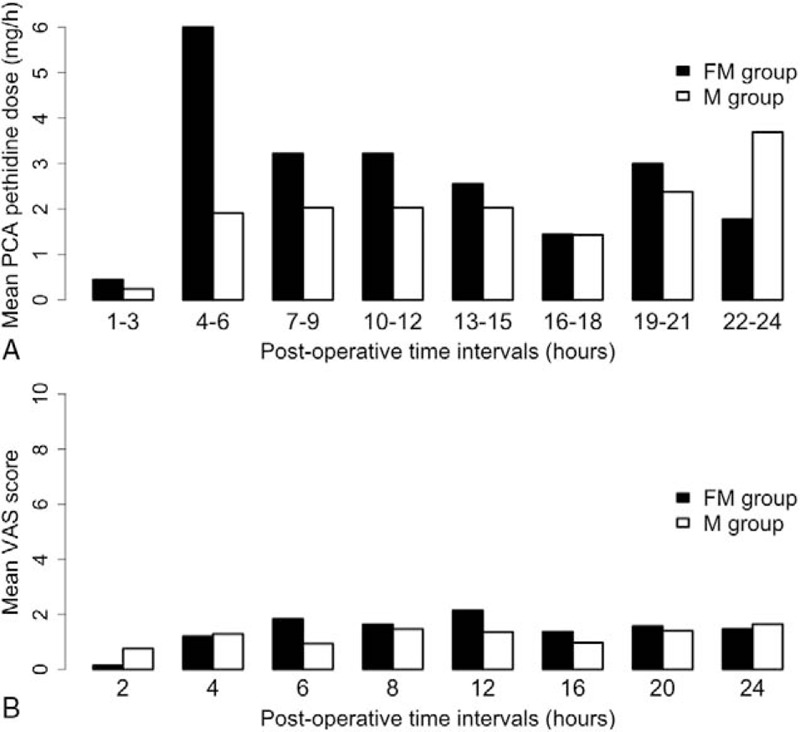
Mean postoperative patient-controlled pethidine consumption (A) and visual analog pain scores (B). Hourly opioid consumption was accumulated into 3-hour intervals to obtain a clear picture of postoperative patient-controlled analgesia requirements and for comparison with the placebo (no intrathecal opioid) group in our previous study^[13]^ (a collation of the results from the present and previous study is provided in Supplementary Fig. 1).

Sensory block up to the T6 dermatome was achieved in all patients. Parturients in the FM group required additional analgesics less often intraoperatively than those in the M group (*P* < .01, relative risk 0.06, 95% CI 0.004–1.04; Table 2). Noninferiority of the new treatment (FM) was confirmed on the basis of mean cumulative patient-controlled pethidine consumption in the 24 hours postoperatively. The 95% CI for the difference between treatment means ranged from −10.0 to 45.7 mg, and was below the prespecified boundary of 50 mg (Table 2, Fig. 2). Postoperative PCA is shown in Fig. 3A.

**Table 2:**
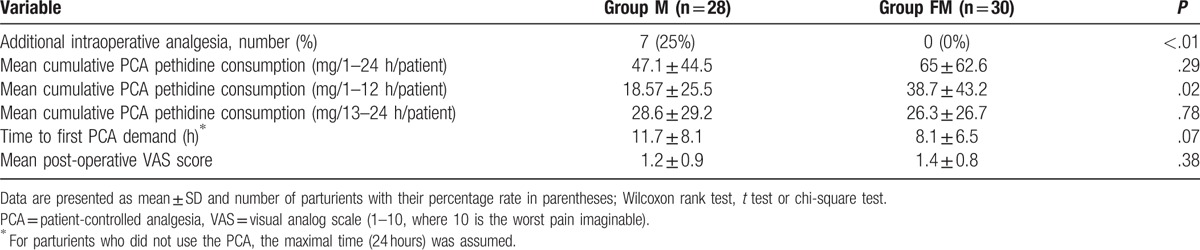
Intraoperative and postoperative analgesia requirements and mean postoperative visual analog pain score.

### 3.3 Secondary outcomes

During the first postoperative time interval (1–12 hours), patients in the FM group required on average more than double the dose of pethidine when compared with the M group (*P* = .02; Table 2). During the second postoperative time interval (13–24 hours), mean cumulative patient-controlled pethidine consumption remained at similar levels in both groups (*P* = .78; Table 2). Duration of effective analgesia and mean VAS scores did not differ between the groups (Fig. 3B, Table 2).

The number of patients experiencing PONV was higher in the FM group (*P* = .01, relative risk 10.3, 95% CI 1.4–74.5; Table 3). The incidence of pruritus was relatively high, but did not differ between the groups. Oversedation and respiratory depression did not occur in any of the patients, and mean oxygen saturation levels were similar (Table 3). There was no difference in Apgar scores between the groups and no clinically important hemodynamic changes occurred in either group intraoperatively or postoperatively.

**Table 3:**
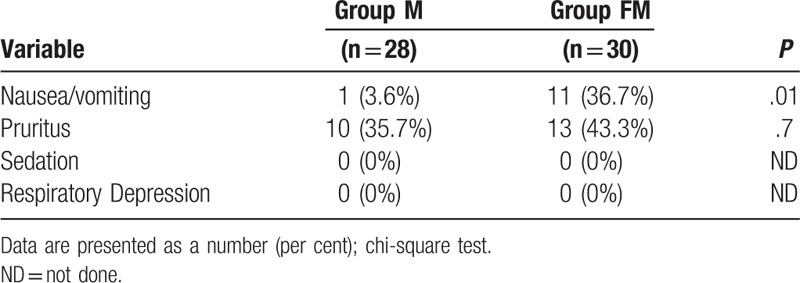
Opioid side effects in the postoperative period.

## 4 Discussion

Our study demonstrates that a combination of intrathecal lipophilic and hydrophilic opioids, such as fentanyl and morphine, can improve perioperative analgesia in patients undergoing CS, but at a cost of more postoperative PONV. Improvement in analgesia was clearly seen intraoperatively. Intrathecal morphine alone, according to some authors, reduces intraoperative discomfort,^[10,24]^ whereas others believe it only starts to work postoperatively.^[31]^ Intraoperative pain has been reported in 18% to 29% of cases after administration of spinal morphine at a dose of 0.1 to 0.2 mg.^[5,6,11,25,31]^ More detailed studies have shown the onset of action of morphine to be 30 to 60 minutes after intrathecal administration in obstetrics^[4]^ and other types of surgery.^[32]^ Given that the duration of CS is usually shorter than 1 hour, the onset of action of morphine during surgery or postoperatively determines the indications for use of spinal fentanyl. The results of our study indicate that morphine does not have an intraoperative analgesic effect, given that 25% of women needed additional intraoperative analgesia. However, the time interval between induction of anesthesia and skin incision in our study was 10 minutes on average. Thus, in the event of a short time interval between spinal anesthesia and the start of surgery, we recommend a combination of the 2 opioids. Other researchers^[25]^ have reached a similar conclusion, and a decrease in intraoperative pain was seen in many studies,^[26–28]^ including in the present study. A search of the literature concerning use of spinal fentanyl in CS revealed that doses much smaller than those used in our study have been equally effective for intraoperative analgesia.^[6,8,25,33]^ This phenomenon was not observed in nonobstetric patients, and a study in an animal model suggested an escalating influence of progesterone on the analgesic effect of lipophilic spinal opioids during pregnancy.^[34]^ In many studies, abolition of intraoperative visceral pain was observed in almost all patients who received spinal fentanyl in doses higher than 10 μg^[7,9,10,24,33,35]^; doses below 10 μg were considered adequate by some authors,^[8]^ but not by others.^[36]^

The mean cumulative patient-controlled pethidine consumption and average VAS scores were not significantly different between the 2 groups in the 24 hours after CS, indicating an overall benefit of combined intrathecal opioids in CS. In contrast, a similar study by Carvalho et al^[6]^ reported no difference in postoperative opioid consumption, but higher VAS scores in the group that received a combination of fentanyl and morphine. However, in our study, when we divided the postoperative period into 2 time intervals, we found a higher demand for patient-controlled pethidine in the FM group during the first 1 to 12 hours, which has been defined previously as the time when the requirement for analgesics after CS is greatest.^[13]^ Sibilla et al^[10]^ similarly noted that only 30% of women who received spinal morphine required additional analgesia during the first 12 hours after CS, whereas 60% of women receiving fentanyl and morphine required additional analgesia in the same time interval. This observation could be explained by the occurrence of acute spinal opioid tolerance. It has been reported that a small dose of spinal fentanyl during CS can result in an increased postoperative demand for intravenous opioids.^[6,37]^ It has also been suggested that an increase in transmission of pain could be linked to 3 mechanisms, that is, reduction of opioid activity at spinal opioid receptors, a reduced effect of endogenous spinal opioids, and an influence on descending pain control pathways.^[37]^ Other mechanistic theories for this phenomenon include the widely held assumption that whereas the onset of action of spinal fentanyl is rapid, the onset of action of morphine administered via the spinal route is slow (which is debatable), such that fentanyl binds to a proportion of spinal opioid receptors before morphine is able to reach them. By the time that the fentanyl is released from these receptors, the proportion of morphine molecules that could potentially bind with them has already been absorbed into the systemic circulation. The remaining concentration of morphine is therefore lower than it would be if morphine was the only substance in the solution from the outset.

The phenomenon of increased demand for opioids after spinal fentanyl was noticed also in the duration of effective analgesia, which was reduced from approximately 12 to 8 hours when opioids were combined. Although this difference was not significant, the trend was quite clear and the result was close to statistical significance (*P* = .07). A similarly short duration of effective analgesia (4–14 hours) when using a combination of fentanyl and morphine has been observed in other studies.^[10,26,27]^ In contrast, the average duration of effective analgesia was 18 to 22 hours in studies when morphine 100 μg was used alone.^[10,27,38–40]^

The mechanism for intrathecal opioid-induced PONV is thought to involve cephalad migration of the opioid in cerebrospinal fluid to opioid receptors in the area postrema.^[28]^ The likelihood of PONV after administration of spinal fentanyl^[8,41]^ or morphine^[21,22,42]^ alone for obstetric purposes has been reported to be relatively low in previous studies, and is consistent with our findings (3.6% for spinal morphine). However, some researchers have reported a higher incidence of PONV after spinal morphine.^[24,40,43,44]^ Unexpectedly, we noted that the incidence of PONV was 37% in patients who received both intrathecal opioids, which is similar to the incidence of 20% to 35% reported elsewhere for use of a combination of opioids.^[3,10,26,27,45]^ One study that reported an increase in PONV when intrathecal opioids were combined suggested that a change in baricity of the opioid solution resulting in hypobaricity, and more cephalad migration of the mixture, might be responsible for the increased incidence of this side effect.^[28]^

A review of the literature shows that addition of morphine to a local anesthetic agent results in an increased incidence of pruritus in the order of 40% to 63%,^[10,24,31,38,39,42]^ which is consistent with our results (36%). A combination of intrathecal opioids, according to some studies, results in an even greater increase in pruritus (67%–87%),^[10,26,45]^ but in other studies, including our present study, the incidence of pruritus was relatively low (37%–48%).^[3,28,45]^

Severe respiratory depression has not been observed after intrathecal administration of morphine at doses of 0.1 to 0.2 mg for CS,^[10,26,38–40]^ and was not seen in our study either. Reviews and other studies on this topic report that respiratory depression after intrathecal opioids is rare and mild in the obstetric population, and that the risk is no higher than after parenteral opioid administration.^[13,24,46]^ It is not clear how a combination of opioids influences the risk of respiratory depression. There has been a report of 1 severe case, which was considered to be associated more with the effect of morphine and not fentanyl, given that it occurred late after administration.^[47]^

There was no relationship between intrathecal opioid administration and neonatal Apgar scores in our study, and this finding is in agreement with other reports.^[8,10,11,31,33]^

In summary, the main contribution of this study to the literature is the finding that a combination of morphine and fentanyl as a supplement to spinal anesthesia for CS provides better intraoperative and postoperative analgesia than does spinal morphine without fentanyl. We have included both periods in our analysis because we believe that the quality of intraoperative analgesia is as important as effective postoperative pain management.

### 4.1 Limitations

We made several assumptions at the beginning of this study. First, we assumed a noninferiority margin of 50 mg on the basis of statistical and clinical reasoning. From the perspective of the intrathecal morphine group, it is almost as high as the mean total pethidine consumption over 24 hours postoperatively. However, the postoperative pethidine requirement in the relevant CS studies has been reported to be 360 to 680 mg in patients who received placebo (ie, no intrathecal opioids).^[48,49]^ In light of these results, our margin of 7% to 14% of this requirement appears to be reasonable. If we have decreased the noninferiority margin to 40 mg, the sample size of 24 patients per group would not exceed the number of patients that we have studied, but the inference of the noninferiority trial would be inconclusive. In this situation, our recommendations about the use of the 2 spinal opioids would have to be weaker. Second, based on the previous studies mentioned earlier in this section, we assumed the difference in occurrence of intraoperative pain between the groups to be 25%. Decreasing the proportion of patients experiencing intraoperative pain in group M to 15% would have increased our sample size by about half of the number of patients that we studied (44 parturients per group). That assumption would mean that our sample size was relatively small. However, sample sizes in comparable studies, also focusing on use of a combination of intrathecal opioids in CS, ranged from 20 to 31 patients per group.^[10,25–28,45]^ Moreover, most studies assessing intraoperative analgesia after addition of spinal fentanyl in CS have used groups containing as few as 5 to 20 patients,^[6–8,33,35,36,50]^ and very few have included greater numbers of patients (29 per group).^[9,10]^

## 5 Conclusions

The combination of intrathecal fentanyl and morphine provides better perioperative analgesia than intrathecal morphine alone and may be recommended in situations where the time from induction of anesthesia to skin incision is short. However, the increase in side effects (PONV) and possibility of acute spinal opioid tolerance after adding intrathecal fentanyl indicates a need for further studies using the same study design and outcome measures, but with lower doses of fentanyl. The practical application of the results of this study could be the use of a combination of lipophilic and hydrophilic spinal opioids as an addition to a local anesthetic agent in spinal anesthesia for CS to increase the patient's comfort level. Based on our results, and those of others, we would recommend using morphine 100 μg and fentanyl 10 to 15 μg.

## Acknowledgments

We are grateful to Professor Jacek Koronacki, Director of the Institute of Computer Science, Polish Academy of Sciences, Warsaw, Poland, for his constructive comments concerning the statistical analysis. We would like to thank Professor Ewa Mayzner-Zawadzka, MD, PhD, DSc, Department of Anesthesiology and Intensive Care, University of Warmia and Mazury, Poland, for mentoring and supervision during the research. We would also like to thank Editage (www.editage.com) for English language editing.

## Supplementary Material

Supplemental Digital Content
