# Supplementary material for: Perioperative analgesia after intrathecal fentanyl and morphine or morphine alone for cesarean section: A randomized controlled study
Source: Medicine (Baltimore). 2017 Dec 1;96(48):e8892. doi: 10.1097/MD.0000000000008892 (PMC5728777; doi:10.1097/MD.0000000000008892)

**Supplementary Figure** **1** (Appendix). Mean post-operative patient-controlled pethidine consumption (A) and visual analog pain scores (B) in the C group (control, no opioid), F group (fentanyl 25░μg),░M group (morphine 100░μg), and FM group (fentanyl 25░μg░+░morphine 100░μg). Hourly opioid consumption was accumulated into 3-h intervals to obtain a clear picture of post-operative patient-controlled analgesia requirements.


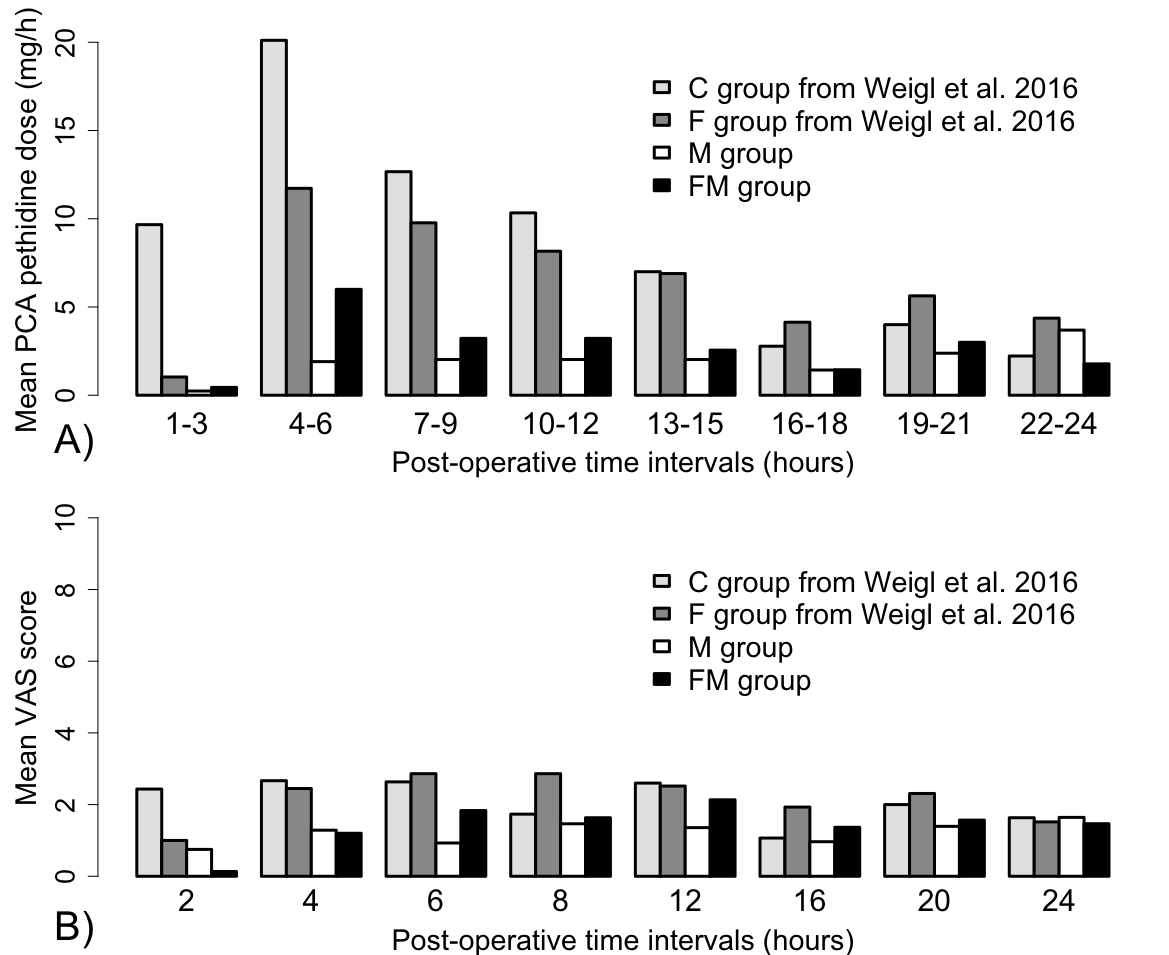

Supplement: Supplemental Digital Content [file medi-96-e8892-s001.doc]
